# Pharmaceutical agents targeting K_ATP_ channel modulate sweet taste sensitivity in mice

**DOI:** 10.1016/j.jphyss.2026.100082

**Published:** 2026-06-05

**Authors:** Chika Sawai, Kuanyu Wang, Kengo Horie, Yoshihiro Mitoh, Hirotaka Ueda, Hiroshi Kamioka, Ryusuke Yoshida

**Affiliations:** a Department of Orthodontics, Graduate School of Medicine, Dentistry and Pharmaceutical Sciences, Okayama University, Japan; b Department of Oral Physiology, Graduate School of Medicine, Dentistry and Pharmaceutical Sciences, Okayama University, Japan; c Faculty of Medicine, Dentistry and Pharmaceutical Sciences, Okayama University, Japan

**Keywords:** Sweet taste receptor, Glucose transporter, Diabetes, Taste disorder, Cephalic phase insulin release

## Abstract

Sweet detection involves at least two mechanisms: a G-protein coupled sweet taste receptor (Tas1r2/Tas1r3) and glucose transporters. As in pancreatic β-cells, glucose transport may lead to closure of ATP-sensitive potassium (K_ATP_) channels. Since expression of K_ATP_ channels in sweet taste cells has been reported, modulation of K_ATP_ channel activity would affect sweet taste sensitivity. Here, we examined the effect of glibenclamide (a K_ATP_ channel closer) and diazoxide (an opener) on mouse taste behavior. Glibenclamide selectively reduced taste sensitivity to glucose without affecting responses to sucrose or sucralose compared to insulin, suggesting selective impairment of the transporter-dependent pathway. In contrast, diazoxide broadly suppressed responses to all tested sweeteners, indicating a generalized effect on sweet detection. Neither drug altered responses to non-sweet taste. These findings suggest that pharmacological modulation of K_ATP_ channel differently influences sweet taste; closers reduce glucose sensitivity whereas openers attenuate response to multiple sweeteners.

## Highlight

• Glibenclamide selectively reduced sensitivity to glucose.
• Diazoxide broadly suppressed responses to all sweeteners.
• Neither agent altered sensitivity to other basic tastes.
• K_ATP_ channel modulators differentially influence sweet taste.

## 1 Introduction

Taste is an important sense that identifies chemical substances in food and regulates feeding behavior as well as nutrient intake. There are five basic tastes: sweet, salty, sour, bitter, and umami. Among them, the sweet taste is indispensable for detecting carbohydrates, a major energy source, and it strongly influences feeding motivation.

Taste sensation begins in taste receptor cells located within the taste buds. Taste bud cells are classified into four types based on their morphological and molecular characteristics [1]. Among them, Type II cells express sweet (Tas1r2/Tas1r3), umami (Tas1r1/Tas1r3), or bitter (Tas2rs) taste receptors and their downstream signaling molecules, thereby functioning as sweet, umami, or bitter taste cells [2], [3], [4], [5], [6]. In contrast, Type III cells express the sour taste receptor (Otop1) and are responsible for detecting acids in the oral cavity [7], [8], [9]. The sweet taste receptor Tas1r2/Tas1r3 is a G protein–coupled receptor that can detect a wide range of sweeteners, including sugars, artificial sweeteners, sweet amino acids, and even some proteins [10]. In addition, recent studies have revealed a sugar-specific sweet taste pathway mediated by glucose transporters such as SGLT1 (sodium–glucose cotransporter 1) and GLUT4 (glucose transporter 4) [11], [12]. Interestingly, these glucose transporters and Tas1r3 are co-expressed with ATP-sensitive K⁺ channels (K_ATP_ channels) [11], [13]. Therefore, similar to pancreatic β-cells, glucose transporters are thought to contribute to taste responses to glucose by influencing the membrane potential of taste cells through the uptake and metabolism of glucose [14], although the detail of mechanisms has not been elucidated in taste cells.

Pancreatic β-cells regulate blood glucose levels by releasing insulin, the master regulator of glucose metabolism, thereby lowering blood glucose. The canonical pathway of insulin release from pancreatic β-cells begins with the uptake of blood glucose via glucose transporters [15]. Glucose is then metabolized through glycolysis and the citrate cycle, resulting in an increased cytosolic ATP/ADP ratio and subsequent closure of K_ATP_ channels. Closure of these K⁺ channels induces membrane depolarization and activates voltage-gated Ca²⁺ channels, which in turn trigger the exocytosis of insulin vesicles [16], [17]. In this way, K_ATP_ channels play a central role in insulin secretion in pancreatic β-cells and are therefore important pharmacological targets for diabetes therapy. K_ATP_ channels are expressed not only in pancreatic β-cells but also in other tissues. For example, in cardiac muscle, K_ATP_ channels contribute to several physiological and pathological processes, including cardioprotection, arrhythmias, and heart failure [18], [19]. Consequently, K_ATP_ channels are also considered pharmacological targets for the treatment of cardiovascular diseases.

K_ATP_ channels are hetero-octameric proteins composed of ion channel components, the inwardly rectifying K⁺ channel (Kir6.x) subunits, and regulatory components, the sulfonylurea receptor (SUR) subunits [20] and are the target for many pharmaceutical agents [21]. Sulfonylurea compounds, such as tolbutamide and glibenclamide, close K_ATP_ channels, thereby inducing depolarization and stimulating insulin release from pancreatic β-cells. These K_ATP_ channel inhibitors are widely used to treat type 2 diabetes [22]. Conversely, K_ATP_ channel openers, such as diazoxide, activate the channels, leading to cell hyperpolarization and reduced insulin secretion from pancreatic β-cells. Accordingly, diazoxide is used to treat hyperinsulinemic hypoglycemia [23]. Although K_ATP_ channel modulators are useful for treating various diseases, including diabetes, administration of these agents may also affect sweet taste sensitivity, since sweet-sensitive taste cells express K_ATP_ channels [11], [13]. Indeed, K_ATP_ channels are involved in the sweet-suppressive effect of leptin [13], [24], [25]. Furthermore, sugar signaling mediated by oral glucose transporters and K_ATP_ channels may contribute to the induction of cephalic phase insulin release (CPIR) [26], [27]. These findings suggest a close relationship between oral sweet sensitivity and K_ATP_ channel activity.

In this study, we investigated the effects of the K_ATP_ channel closer glibenclamide and the opener diazoxide on sweet taste sensitivity in mice. We first compared blood glucose levels after administration of saline, glibenclamide, diazoxide or insulin. Then we analyzed short-term licking behaviors in response to various taste solutions in the insulin vs. glibenclamide group and in the saline vs. diazoxide group according to their blood glucose levels. We also examined whether administration of glibenclamide or diazoxide alters conditioned taste aversion (CTA) behaviors to a sweet tastant. Our results suggest that both the K_ATP_ channel closer and opener influence taste responses to sweeteners in mice, with differences in sensitivity depending on the type of sweetener.

## 2 Methods

### 2.1 Animals

All experimental procedures were conducted in accordance with the National Institutes of Health Guide for the Care and Use of Laboratory Animals and were approved by the Committee for Laboratory Animal Care and Use at Okayama University, Japan. The study subjects were adult male C57BL/6 J (B6) mice (CLEA Japan, Tokyo, Japan) older than eight weeks. Mice were housed under a 12:12-hour light–dark cycle (lights on from 08:00–20:00) with ad libitum access to tap water and standard food pellets (MF, Oriental Yeast Co., Tokyo, Japan) prior to the experiments.

### 2.2 Blood glucose measurement

Blood glucose levels were measured 15 min after intraperitoneal injection of diazoxide (10 mg/Kg body weight), glibenclamide (10 mg/Kg body weight), insulin (0.1 mg/Kg body weight) or saline [27], [28], [29]. The volume of solution injected was 1/100 of the body weight (200 µL for a 20 g mouse). Mice were fasted for 12 h prior to measurement. Blood samples were collected from the tail tip, and glucose concentrations were measured using a portable blood glucose meter (Glutest Mint II, Sanwa Kagaku Kenkyusho, Aichi, Japan). B6 mice were used for diazoxide (n = 5), glibenclamide (n = 5), insulin (n = 5) and saline administration (n = 5).

### 2.3 Short-term lick test

Short-term lick responses to various tastants were measured according to a previously described method using binary mixture of sweet or umami and bitter taste [30]. Using this method, clear concentration dependent responses to preferable solution could be obtained and the effect of specific treatment on sweet or umami sensitivity could be analyzed [31]. B6 mice were divided into four groups: saline (n = 8), glibenclamide (n = 8), diazoxide (n = 8) and insulin (n = 8). Each mouse was housed individually throughout the experiment. On the first day of training, mice were water-deprived for 12 h and then placed in a test cage with free access to deionized water for 1 h. During training sessions (days 2–5), mice were trained to drink deionized water on an interval schedule consisting of 5 s water presentation periods alternating with 10 s intertrial intervals. From day 6, the number of licks for each tastant and for deionized water was recorded during the first 5 s following the initial lick using a lick meter (Yutaka Electronics Co., Gifu, Japan). The taste solutions used in this experiment were as follows: 30–1000 mM sucrose + 0.3 mM quinine-HCl (QHCl) (Suc+Q), 30–1000 mM glucose + 0.3 mM QHCl (Glc+Q), 0.3–10 mM sucralose + 0.3 mM QHCl (Sucra+Q), 1–100 mM HCl, 30–1000 mM NaCl, 0.01–3 mM QHCl, and 10–300 mM monosodium glutamate (MSG) + 0.3 mM QHCl (MSG+Q). On each day, one tastant was tested at various concentrations, beginning 15 min after intraperitoneal administration of saline, glibenclamide (10 mg/Kg body weight), diazoxide (10 mg/Kg body weight), or insulin (0.1 mg/Kg body weight). The volume of solution injected was 1/100 of the body weight (200 µL for a 20 g mouse). All taste tests were completed within 3 h of drug administration to minimize variability due to metabolic degradation or systemic effects. For preferred solutions (Suc+Q, Glc+Q, Sucra+Q, MSG+Q), mice were deprived of both food and water for 12 h prior to testing. On the test day, these solutions were presented first in descending concentration order (from the highest concentration to deionized water), followed by randomized presentation in subsequent trials. For aversive solutions (NaCl, QHCl, HCl), mice were water-deprived for 12 h before testing. On test days, these solutions were presented in ascending concentration order (from deionized water to the highest concentration), followed by randomized presentation in subsequent trials. Each solution was tested in at least three lick trials, and the mean lick count was used for data analysis.

### 2.4 CTA test

Lick responses to various tastants were assessed following aversive conditioning to a sucrose solution [32]. B6 mice were divided into four groups: saline (n = 7), glibenclamide (n = 8), diazoxide (n = 9), and insulin (n = 8). Each mouse was housed individually throughout the experiment. The lick training procedure was the same as that described for the short-term lick test except for the time of water presentation (10 s in CTA test). On day 6, mice were given access to 100 mM sucrose [conditioned stimulus (CS)] during 10 s presentation periods for more than 20 trials (10 s licking CS/trial). Immediately afterward, they received an intraperitoneal injection of 0.24 M LiCl [230 mg/Kg body weight, unconditioned stimulus (US)] to induce gastrointestinal malaise. Day 7 was designated as a recovery period without treatment. From days 8–11, CTA testing was conducted 15 min after intraperitoneal administration of saline, glibenclamide (10 mg/Kg body weight), diazoxide (10 mg/Kg body weight), or insulin (0.1 mg/Kg body weight). The volume of solution injected was 1/100 of the body weight (200 µL for a 20 g mouse). During the test period, mice were presented with various tastants and deionized water in randomized order, continuing until they ceased licking deionized water within 7 s after their first lick in a given trial. In each trial, the number of licks was recorded during the first 10 s following the initial lick. Tastants used for CTA testing included: 30–1000 mM sucrose (Suc), 30–1000 mM glucose (Glc), 0.3–10 mM sucralose (Sucra), 1–30 mM HCl, 30–1000 mM NaCl, 0.01–1 mM QHCl, and 10–300 mM monopotassium glutamate (MPG).

### 2.5 Chemicals

Diazoxide (Sigma-Aldrich, MO, USA), glibenclamide (Fujifilm Wako Pure Chemical, Osaka, Japan), recombinant human insulin (Fujifilm Wako Chemical), sucralose (Sigma-Aldrich), MPG (Thermo Fisher Scientific, MA, USA) were used. Other chemicals were purchased from Nacalai Tesque (Kyoto, Japan).

### 2.6 Data analysis

For statistical analysis of blood glucose levels, one-way ANOVA and a post hoc Tukey-HSD test was performed. For statistical analysis of lick responses, repeated two-way ANOVA was performed to compare the concentration of taste solution, treatment, and interaction effect. All statistical analyses were conducted by Jamovi software (version 2.3.28) and significance was determined at P-values < 0.05.

## 3 Results

### 3.1 Blood glucose measurement

**Fig. 1:**
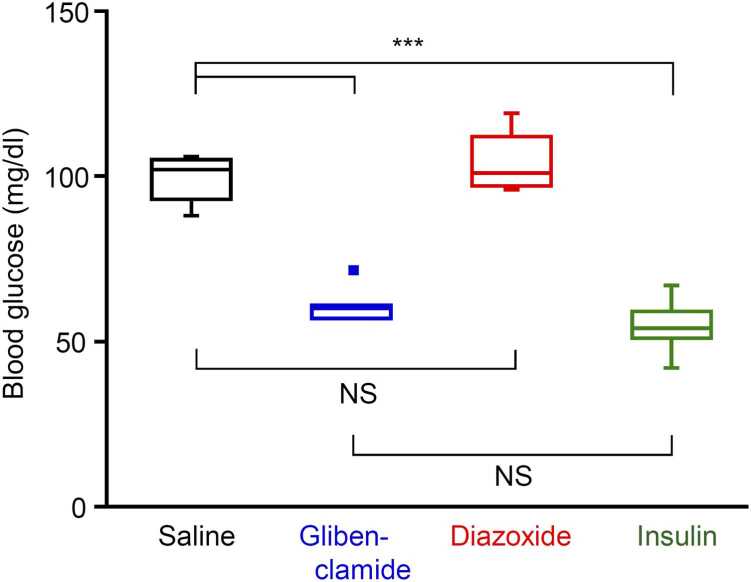
Measurement of blood glucose levels. Blood glucose levels 15 min after intraperitoneal administration of saline, glibenclamide (10 mg/Kg body weight), diazoxide (10 mg/Kg body weight) or insulin (0.1 mg/Kg body weight). The volume of solution injected was 1/100 of the body weight (200 µL for a 20 g mouse). In these box plots, the box indicates the 25th and 75th percentiles; line across the box, the median; the dot, outlier; and whiskers, maximum and minimum values. ***: P < 0.001, NS: not significantly different, post hoc Tukey test.

We first measured blood glucose levels 15 min after administration of saline, glibenclamide or diazoxide since glibenclamide would reduce blood glucose levels via insulin secretion (Fig. 1). No significant difference was observed between the saline- and diazoxide-administrated groups, whereas administration of glibenclamide significantly decreased blood glucose levels compared with the saline group. Administration of insulin (0.1 mg/Kg body weight) also significantly decreased blood glucose levels, similar to glibenclamide administration. Therefore, in the following experiments, we compared the glibenclamide group with the insulin group and the diazoxide group with the saline group.

### 3.2 Effect of glibenclamide

**Fig. 2:**
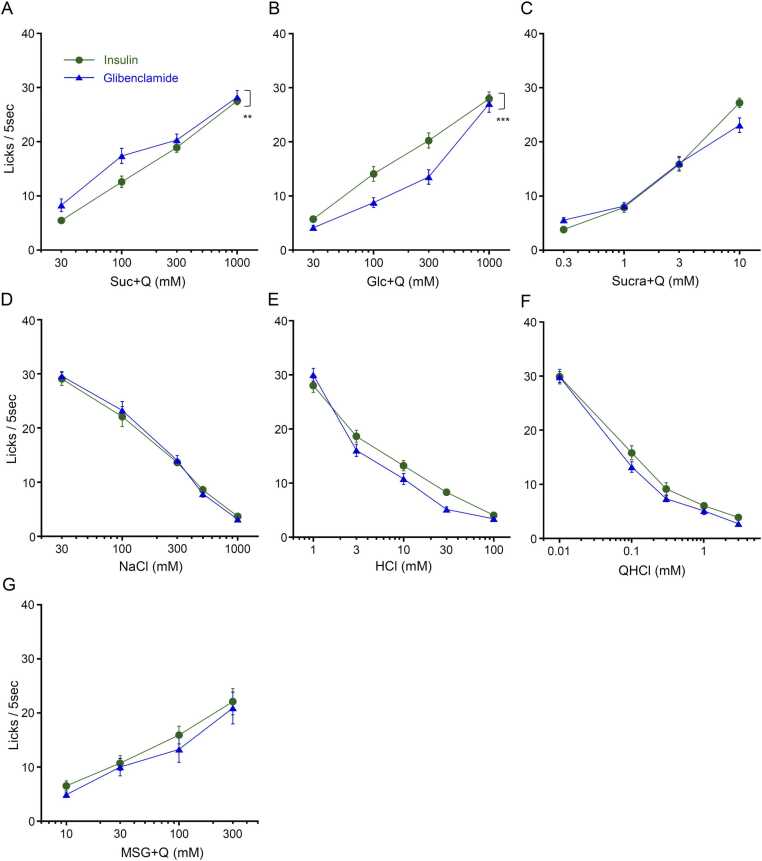
Effects of glibenclamide on licking responses to taste stimuli in short-term licking tests. Number of licking responses to 30–1000 mM Suc + Q (A), 30–1000 mM Glc + Q (B), and 0.3–10 mM Sucra + Q (C), 30–1000 mM NaCl (D), 1–100 mM HCl (E), 0.01–3 mM QHCl (F), and 10–300 mM MSG + Q (G). Green circles: Insulin group (0.1 mg/Kg body weight, n = 8), Blue triangles: Glibenclamide group (10 mg/Kg body weight, n = 8). Values are mean ± standard error of the mean (SEM). Statistical differences were analyzed by repeated two-way ANOVA (**: P < 0.01, ***: P < 0.001).

**Fig. 3:**
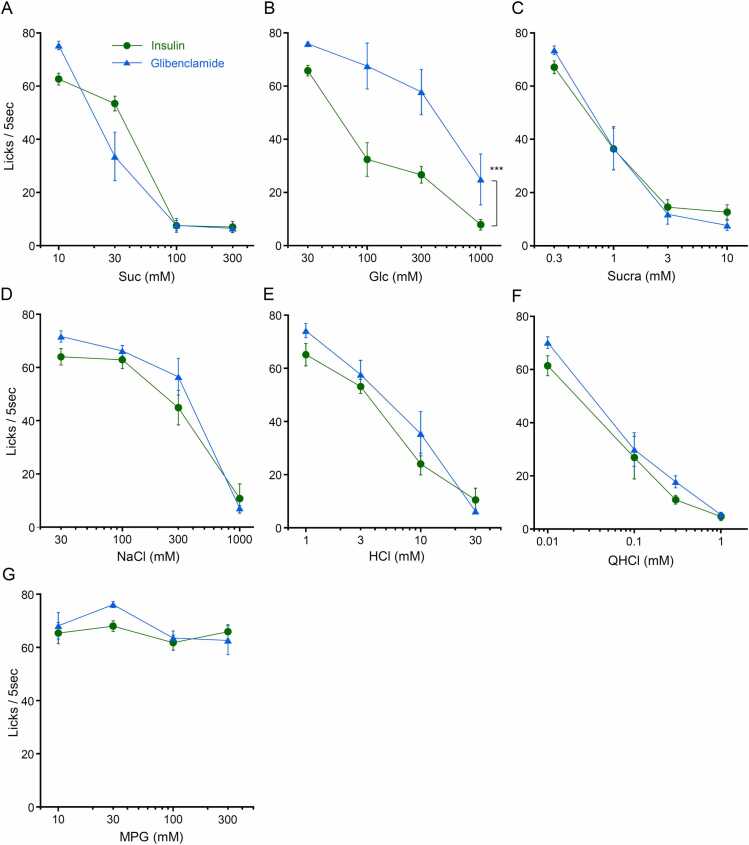
Effects of glibenclamide on licking responses to taste stimuli in CTA tests. Number of licking responses to 10–300 mM Suc (A), 30–1000 mM Glc (B), and 0.3–10 mM Sucra (C), 30–1000 mM NaCl (D), 1–30 mM HCl (E), 0.01–1 mM QHCl (F), and 10–300 mM MPG (G). Green circles: Insulin group (0.1 mg/Kg body weight, n = 8), Blue triangles: Glibenclamide group (10 mg/Kg body weight, n = 8). Values represent mean ± SEM. Statistical differences were analyzed by repeated two-way ANOVA (***: P < 0.001).

We performed short-term lick tests in B6 mice treated with insulin or glibenclamide. Mice administered insulin showed concentration-dependent increases in lick responses to Suc + Q, Glc + Q, Sucra + Q, and MSG + Q (Fig. 2A–C, G). Similarly, mice administered glibenclamide also exhibited concentration-dependent increases in lick responses to these tastants (Fig. 2A–C, G). A comparison between the insulin and glibenclamide groups revealed that glibenclamide significantly increased lick responses to Suc + Q and reduced lick responses to Glc + Q (Fig. 2A,B, Table 1). Both groups showed similar concentration-dependent decreases in lick responses to NaCl, HCl, and QHCl (Fig. 2D–F, Table 1). We further examined the effect of glibenclamide on taste sensitivity using a CTA test. In this assay, B6 mice were conditioned to avoid 100 mM Suc. Both the insulin and glibenclamide groups showed strong aversion to 100 mM Suc (Fig. 3A). These mice also exhibited aversion to other sweet compounds, including Glc and Sucra (Fig. 3B, C). A comparison between groups revealed that the glibenclamide group showed weaker aversion to glucose, but not to Suc or Sucra, compared with the insulin group (Fig. 3A–C, Table 2). Lick responses to other tastants, including NaCl, HCl, QHCl, and MPG, did not differ significantly between the two groups (Fig. 3D–G, Table 2). These findings suggest that glibenclamide primarily suppresses taste sensitivity to glucose. Lick responses of insulin group were almost similar to those of saline group (Supplemental Figure 1 and 2).

**Table 1:** Two-way ANOVA results for the effect of glibenclamide in short-term lick test (Fig. 2).

**Tastant**	**Effect**	**Degree of Freedom**	**F Value**	***p*****Value**
Suc + Q	treatment	1,14	9.224	0.009**
	concentration	3,42	155.2	< 0.001***
	interaction	3,42	1.641	0.194
Glc + Q	treatment	1,14	10.81	0.005**
	concentration	3,42	179.1	< 0.001***
	interaction	3,42	3.91	0.015*
Sucra + Q	treatment	1,14	0.409	0.533
	concentration	3,42	208.4	< 0.001***
	interaction	3,42	4.001	0.014*
NaCl	treatment	1,14	0.032	0.860
	concentration	4,56	254.7	< 0.001***
	interaction	4,56	0.353	0.841
HCl	treatment	1,14	3.373	0.088
	concentration	4,56	292.6	< 0.001***
	interaction	4,56	3.225	0.019*
QHCl	treatment	1,14	3.96	0.067
	concentration	4,56	313.2	< 0.001***
	interaction	4,56	0.628	0.645
MSG + Q	treatment	1,14	0.468	0.505
	concentration	3,42	67.9	< 0.001***
	interaction	3,42	0.252	0.860

**Table 2:** Two-way ANOVA results for the effect of glibenclamide in the CTA test (Fig. 3).

**Tastant**	**Effect**	**Degree of Freedom**	**F Value**	***p*****Value**
Suc	treatment	1,14	0.662	0.429
	concentration	3,42	117.6	< 0.001***
	interaction	3,42	5.82	0.002**
Glc	treatment	1,14	24.4	< 0.001***
	concentration	3,42	29.3	< 0.001***
	interaction	3,42	1.999	0.129
Sucra	treatment	1,14	0.010	0.923
	concentration	3,42	68.2	< 0.001***
	interaction	3,42	0.535	0.661
NaCl	treatment	1,14	2.061	0.173
	concentration	3,42	83.9	< 0.001***
	interaction	3,42	1.216	0.316
HCl	treatment	1,14	2.423	0.142
	concentration	3,42	72.1	< 0.001***
	interaction	3,42	1.157	0.338
QHCl	treatment	1,14	2.243	0.142
	concentration	3,42	89.1	< 0.001***
	interaction	3,42	0.418	0.741
MPG	treatment	1,14	0.412	0.531
	concentration	3,42	4.696	0.006**
	interaction	3,42	1.500	0.531

### 3.3 Effect of diazoxide

**Fig. 4:**
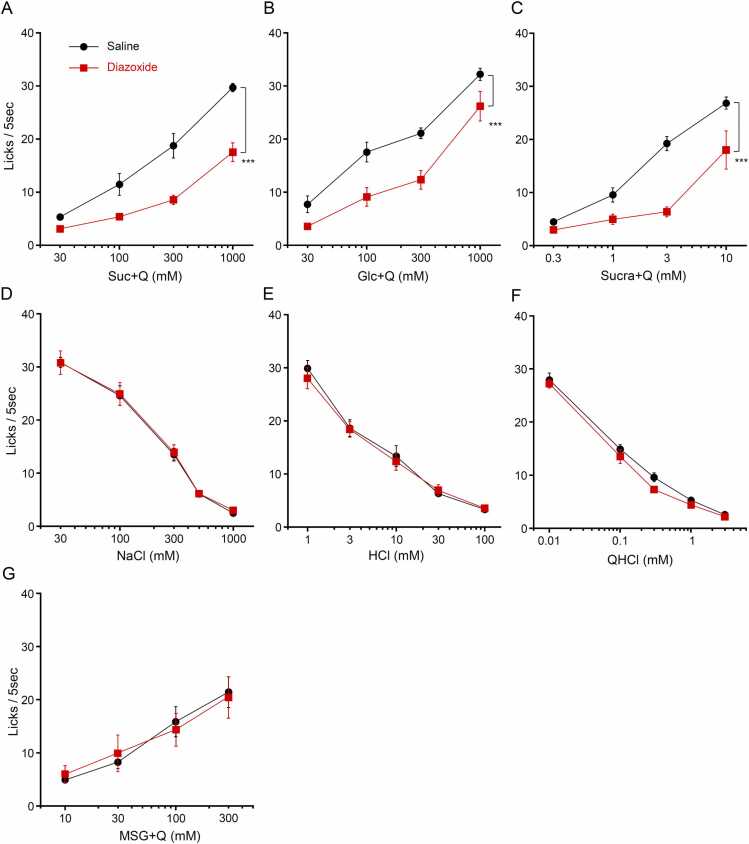
Effects of diazoxide on licking responses to taste stimuli in short-term licking tests. Number of licking responses to 30–1000 mM Suc + Q (A), 30–1000 mM Glc + Q (B), 0.3–10 mM Sucra + Q (C), 30–1000 mM NaCl (D), 1–100 mM HCl (E), 0.01–3 mM QHCl (F), and 10–300 mM MSG + Q (G). Black circles: Saline group (n = 7), Red squares: Diazoxide group (10 mg/Kg body weight, n = 9). Values represent mean ± SEM. Statistical differences were analyzed by repeated two-way ANOVA (***: P < 0.001).

**Fig. 5:**
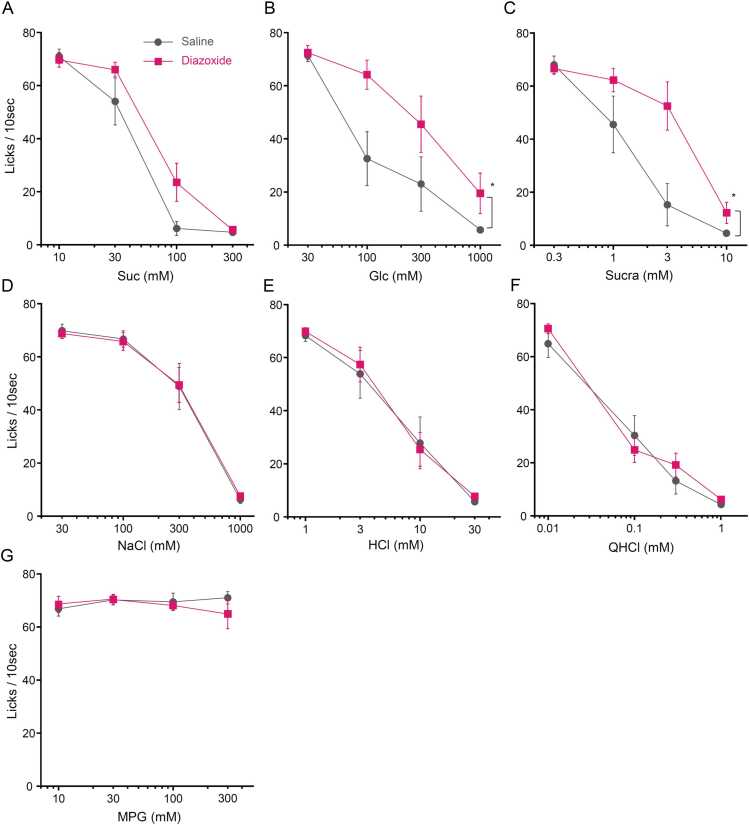
Effects of diazoxide on licking responses to taste stimuli in CTA tests Licking responses to 10–300 mM Suc (A), 30–1000 mM Glc (B), and 0.3–10 mM Sucra (C), 30–1000 mM NaCl (D), 1–30 mM HCl (E), 0.01–1 mM QHCl (F), and 10–300 mM MPG (G). Black circles: Saline group (n = 7), Red squares: Diazoxide group (10 mg/Kg body weight, n = 9). Values represent mean ± SEM. Statistical differences were analyzed by repeated two-way ANOVA (*: P < 0.05).

Next, we assessed the effect of diazoxide on mouse taste responses. In short-term lick tests, the diazoxide group exhibited concentration-dependent increases in lick responses to Suc + Q, Glc + Q, Sucra + Q, and MSG + Q (Fig. 4A–C, G). Unlike glibenclamide, however, diazoxide significantly decreased lick responses to all sweeteners tested compared with saline-treated mice (Fig. 4A–C, Table 3). Lick responses to responses to NaCl, HCl, QHCl, and MSG + Q were unchanged by diazoxide treatment (Fig. 4D–F, Table 3), suggesting that its effects were specific to sweet taste responses. In CTA tests, diazoxide-treated mice conditioned to avoid 100 mM Suc exhibited higher lick responses to sweet solutions than saline-treated mice (Fig. 5A–C). Statistically significant differences between the diazoxide and saline groups were observed for Glc and Sucra (Table 4). Lick responses to NaCl, HCl, QHCl, and MPG did not differ significantly between groups (Fig. 5D–G, Table 4). These results suggest that diazoxide suppresses behavioral lick responses to a range of sweeteners.

**Table 3:** Two-way ANOVA results for the effect of diazoxide in short-term lick test (Fig. 4).

**Tastant**	**Effect**	**Degree of Freedom**	**F Value**	***p*****Value**
Suc + Q	treatment	1,14	32.5	< 0.001***
	concentration	3,42	111.7	< 0.001***
	interaction	3,42	7.78	< 0.001***
Glc + Q	treatment	1,14	17.98	< 0.001***
	concentration	3,42	99.0	< 0.001***
	interaction	3,42	1.205	0.320
Sucra + Q	treatment	1,14	25.9	< 0.001***
	concentration	3,42	64.9	< 0.001***
	interaction	3,42	5.96	0.002**
NaCl	treatment	1,14	0.031	0.864
	concentration	4,56	245.2	< 0.001***
	interaction	4,56	0.027	0.999
HCl	treatment	1,14	0.087	0.773
	concentration	4,56	163.6	< 0.001***
	interaction	4,56	0.385	0.818
QHCl	treatment	1,14	0.248	0.626
	concentration	4,56	121.5	< 0.001***
	interaction	4,56	1.159	0.337
MSG + Q	treatment	1,14	0.174	0.683
	concentration	3,42	0.435	0.729
	interaction	3,42	0.907	0.446

**Table 4:** Two-way ANOVA results for the effect of diazoxide in the CTA test (Fig. 5).

**Tastant**	**Effect**	**Degree of Freedom**	**F Value**	***p*****Value**
Suc	treatment	1,14	3.47	0.084
	concentration	3,42	126.5	< 0.001***
	interaction	3,42	2.408	0.081
Glc	treatment	1,14	6.585	0.022*
	concentration	3,42	29.0	< 0.001***
	interaction	3,42	1.976	0.132
Sucra	treatment	1,14	6.958	0.019*
	concentration	3,42	45.0	< 0.001***
	interaction	3,42	4.675	0.007**
NaCl	treatment	1,14	0.000	0.991
	concentration	3,42	98.2	< 0.001***
	interaction	3,42	0.040	0.989
HCl	treatment	1,14	0.066	0.801
	concentration	3,42	57.8	< 0.001***
	interaction	3,42	0.120	0.948
QHCl	treatment	1,14	3.96	0.067
	concentration	3,42	313.2	< 0.001***
	interaction	3,42	0.628	0.645
MPG	treatment	1,14	0.468	0.505
	concentration	3,42	67.9	< 0.001***
	interaction	3,42	0.252	0.860

## 4 Discussion

K_ATP_ channel subunits SUR1 and Kir6.1 are expressed in taste bud cells [11]. In particular, SUR1 has been reported to be co-expressed with Tas1r3 [11], [13], suggesting that sweet-sensitive taste cells possess functional K_ATP_ channels. Consistent with these findings, our behavioral experiments demonstrated that a K_ATP_ channel closer (glibenclamide) and opener (diazoxide) specifically affected sweet taste sensitivity in mice. Pharmaceutical agents targeting K_ATP_ channels have clinical significance in the treatment of various diseases, including type 2 diabetes [22] and hyperinsulinemic hypoglycemia [23], and are also being explored for potential use in conditions such as pulmonary hypertension [33] and migraine [34]. Although the expression of K_ATP_ channel subunits in human taste tissue has not yet been characterized, clinical treatments involving K_ATP_ channel inhibitors or openers could potentially influence sweet taste sensitivity in humans. At least, changes in sense of taste were reported as a side effect of hyperinsulinism Global Registry participants currently taking diazoxide although details of changes in taste sensation were not described [35].

In the taste buds, two distinct systems are involved in the detection of sweet substances [14]. The sweet taste receptor Tas1r2/Tas1r3 is a G-protein–coupled receptor capable of detecting a wide variety of sweet compounds, including sugars, artificial sweeteners, sweet amino acids, and even sweet proteins. Upon binding of sweet compounds to Tas1r2/Tas1r3, an intracellular signaling pathway involving gustducin, phospholipase C β2 (Plcβ2), and transient receptor potential channel M5 (Trpm5) is activated, leading to the generation of action potentials in sweet-sensitive taste cells [2], [3], [5], [36]. The other pathway relies on glucose transporters [14]. In this mechanism, glucose is transported into the cell via glucose transporters and subsequently metabolized to generate ATP. The resulting increase in intracellular ATP leads to K_ATP_ channel closure, depolarizing sweet-sensitive taste cells. Therefore, glucose is the primary substrate for activating the glucose transporter–dependent pathway.

**Fig. 6:**
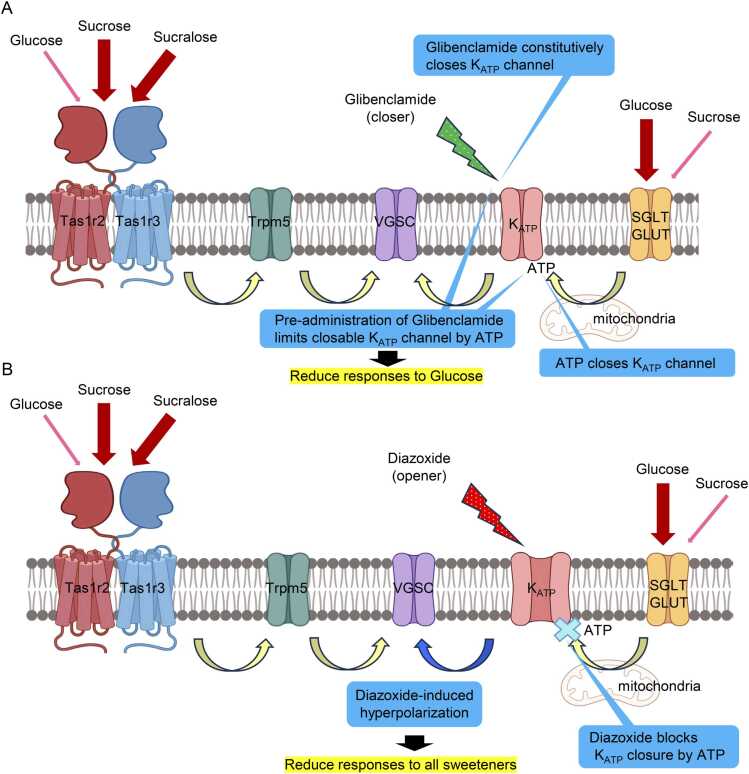
Hypothetical diagram of K_ATP_ channels and sweet taste sensitivity. Two types of sweet taste systems exist: Tas1r3-dependent sweet taste receptors and glucose transporters. K_ATP_ channels are expressed in these sweet-sensitive cells. Glibenclamide inhibits the glucose transporter pathway and suppresses glucose responses mediated by glucose transporters, but has little effect on the activation of taste cells by Tas1r3-dependent receptors (A). In contrast, diazoxide opens K_ATP_ channels, inhibiting the glucose transporter pathway while simultaneously suppressing depolarization via Tas1r3-dependent receptors by inducing cellular hyperpolarization (B).

In this study, we found that glibenclamide specifically reduced lick responses to glucose solution (Fig. 2, Fig. 3). Since glibenclamide is a K_ATP_ channel inhibitor, it may interfere with the glucose transporter–dependent pathway, which also inhibits K_ATP_ channel (Fig. 6A). Pre-administration of glibenclamide would close K_ATP_ channels on the sweet sensitive taste cells. Under this condition, even if glucose is taken up into taste cells and ATP is produced, the generated ATP would affect a limited number of K_ATP_ channels, as these channels are already closed by glibenclamide. Therefore, depolarization induced via glucose transporter–dependent pathway might be smaller in the presence of glibenclamide than in its absence. Thus, administration of glibenclamide could suppress glucose responses by disrupting the glucose transporter–dependent pathway. In pancreatic β-cells, each of glucose and glibenclamide induced similar levels of insulin release, and co-application of glucose and glibenclamide also resulted in a comparable level of insulin release [37], indicating that their combined effects are not necessarily additive. K_ATP_ channel closure might have some effects on the Tas1r3-dependent pathway, as glibenclamide administration affected licking responses to sucrose, which is primarily recognized via Tas1r3 receptors. Sensitivity to low concentrations of sucrose may be slightly enhanced, as responses to 30 and 100 mM sucrose were slightly increased in the lick tests, and responses to 30 mM sucrose were decreased in the CTA test. This may occur because depolarization shifts the membrane potential closer to threshold, thereby facilitating action potential generation even with weak stimuli.

In contrast, diazoxide decreased taste sensitivity to all sweeteners tested (Fig. 4, Fig. 5). Activation of K_ATP_ channels by diazoxide is expected to hyperpolarize sweet-sensitive taste cells. Under this condition, depolarization normally elicited via the Tas1r2/Tas1r3–mediated pathway could be counteracted by K_ATP_ channel–mediated hyperpolarization (Fig. 6B). Furthermore, the glucose transporter–dependent pathway, which relies on K_ATP_ channel closure for depolarization, may be disrupted by forced channel opening following diazoxide administration. Consequently, taste sensitivity to various sweeteners was reduced. Previous studies have shown that leptin suppresses sweet taste responses via activation of K_ATP_ channels [13], [24], [25]. Consistent with our findings, leptin likely activates K_ATP_ channels in sweet-sensitive taste cells, leading to suppressed responses to a variety of sweet compounds. These observations suggest that K_ATP_ channel opening generally reduces the excitability of sweet-sensitive taste cells, resulting in decreased sensitivity to multiple sweeteners.

In this study, we investigated the effects of K_ATP_ channel modulators on taste sensitivity by examining taste behavior in mice. Based on these observations, we proposed a hypothetical model of sweet taste modulation by these modulators (Fig. 6). However, confirmation of this model will require studies at the cellular level, such as perforated patch-clamp recordings to measure the membrane potential (Vm) of taste bud cells [38]. Although performing such electrophysiological experiments is technically challenging for our group, future studies using these techniques will help elucidate the cellular mechanisms underlying the effects of K_ATP_ channel modulators.

K_ATP_ channels in sweet-sensitive taste cells likely serve at least two functions: the generation of glucose responses and the modulation of sweet responses. K_ATP_ channel–mediated glucose responses in taste cells play an important role in eliciting the CPIR [26], [27]. Although Sur1 knockout mice, which presumably lack functional K_ATP_ channels, have been reported to exhibit glucose responses similar to wild-type mice, our data suggest that modulation of K_ATP_ channel activity does affect glucose responses. These discrepancies may arise from differences in behavioral testing protocols, such as the use of sweet tastants versus sweet–bitter mixtures, or standardized lick ratios versus total lick counts. Glendinning et al. reported that K_ATP_ channel modulators influence the magnitude of CPIR [26], supporting the idea that K_ATP_ channel activity in taste cells contributes to insulin secretion initiated by oral glucose detection. Regarding modulation of sweet taste responses, diazoxide has been shown to suppress taste cell responses to sweeteners, similar to the effect of leptin application. Furthermore, glibenclamide significantly blocked the sweet-suppressive effect of leptin [13]. This inhibitory effect of leptin on sweet taste may help regulate energy intake, acting in concert with its central effects on appetite control.

In this study, both glibenclamide and diazoxide were administered via intraperitoneal injection. This procedure may have induced systemic effects, such as changes in blood insulin or glucose levels, which could potentially influence the animals’ sweet taste sensitivity. In rats, blood glucose levels have been reported to affect perceived sweet intensity [39]. In contrast, a human study demonstrated that sweet taste sensitivity correlates with blood leptin levels, but not with insulin or glucose levels [40]. It remains unclear whether systemic changes induced by K_ATP_ channel modulators directly affect taste sensitivity. Therefore, further studies are needed to clarify the mechanisms underlying changes in sweet taste sensitivity following administration of K_ATP_ channel modulators.

## 5 Conclusions

This study demonstrates that pharmacological modulation of K_ATP_ channels influences sweet taste sensitivity in mice, although the effects depend on both the type of sweetener and the direction of K_ATP_ channel modulation. Diazoxide suppressed responses to all tested sweeteners, including sucrose, glucose, and sucralose, suggesting that membrane hyperpolarization broadly interferes with sweet taste signaling. In contrast, glibenclamide reduced taste sensitivity to glucose. These findings indicate that multiple mechanisms contribute to sweet taste detection, including the canonical Tas1r2/Tas1r3 receptor pathway and a transporter- and metabolism-linked pathway involving glucose uptake and K_ATP_ channel closure. Furthermore, our results suggest that the clinical use of K_ATP_ channel–targeting agents, such as glibenclamide and diazoxide, may influence taste perception—particularly sweet taste—which could affect dietary behavior and food enjoyment in patients.

## CRediT authorship contribution statement

**Hiroshi Kamioka:** Writing – review & editing, Validation, Supervision, Investigation, Formal analysis. **Hirotaka Ueda:** Writing – review & editing, Validation, Supervision, Investigation, Formal analysis. **Yoshihiro Mitoh:** Writing – review & editing, Validation, Supervision, Investigation, Formal analysis. **Kengo Horie:** Writing – review & editing, Validation, Supervision, Methodology, Formal analysis. **Kuanyu Wang:** Writing – review & editing, Visualization, Validation, Methodology, Investigation, Data curation. **Chika Sawai:** Writing – review & editing, Writing – original draft, Visualization, Validation, Methodology, Investigation, Formal analysis, Data curation. **Ryusuke Yoshida:** Writing – review & editing, Writing – original draft, Visualization, Validation, Supervision, Project administration, Methodology, Investigation, Funding acquisition, Formal analysis, Data curation, Conceptualization.

## Consent for publication

Not applicable.

## Ethics approval and consent to participate

All animal experiments were performed in accordance with the National Institutes of Health Guide for the Care and Use of Laboratory Animals and approved by the Committee for Laboratory Animal Care and Use and the local ethics committee at Okayama University, Japan.

## Funding

This work was supported by Japan Society for the Promotion of Science KAKENHI grants 21H03106, 23K21484, 24K22186 (R.Y.). The funding source had no role in the design of the study, in collection, analysis, and interpretation of data, or in writing the manuscript

## Declaration of Competing Interest

The authors declare that they have no known competing financial interests or personal relationships that could have appeared to influence the work reported in this paper.

## Data Availability

All data supporting the findings of this study are available from the corresponding author upon request.

## Appendix A Supplementary material

Supplemental Figure 1. Licking responses to taste stimuli in short-term licking tests. Number of licking responses to 30–1000 mM Suc + Q (A), 30–1000 mM Glc + Q (B), and 0.3–10 mM Sucra + Q (C), 30–1000 mM NaCl (D), 1–100 mM HCl (E), 0.01–3 mM QHCl (F), and 10–300 mM MSG + Q (G). Green circles: Insulin group (0.1 mg/Kg body weight, n = 8), Black circles: Saline group (n = 7). Values are mean ± SEM

Supplemental Figure 2. Licking responses to taste stimuli in CTA tests. Number of licking responses to 10–300 mM Suc (A), 30–1000 mM Glc (B), and 0.3–10 mM Sucra (C), 30–1000 mM NaCl (D), 1–30 mM HCl (E), 0.01–1 mM QHCl (F), and 10–300 mM MPG (G). Green circles: Insulin group (0.1 mg/Kg body weight, n = 8), Blue triangles: Black circles: Saline group (n = 7). Values represent mean ± SEM

## Footnotes

Appendix A Supplementary data associated with this article can be found in the online version at doi:10.1016/j.jphyss.2026.100082.
