# Supplementary figures and images for: Pharmaceutical agents targeting KATP channel modulate sweet taste sensitivity in mice
Source: J Physiol Sci. 2026 Jun 5;76(2):100082. doi: 10.1016/j.jphyss.2026.100082 (PMC13279012; doi:10.1016/j.jphyss.2026.100082)

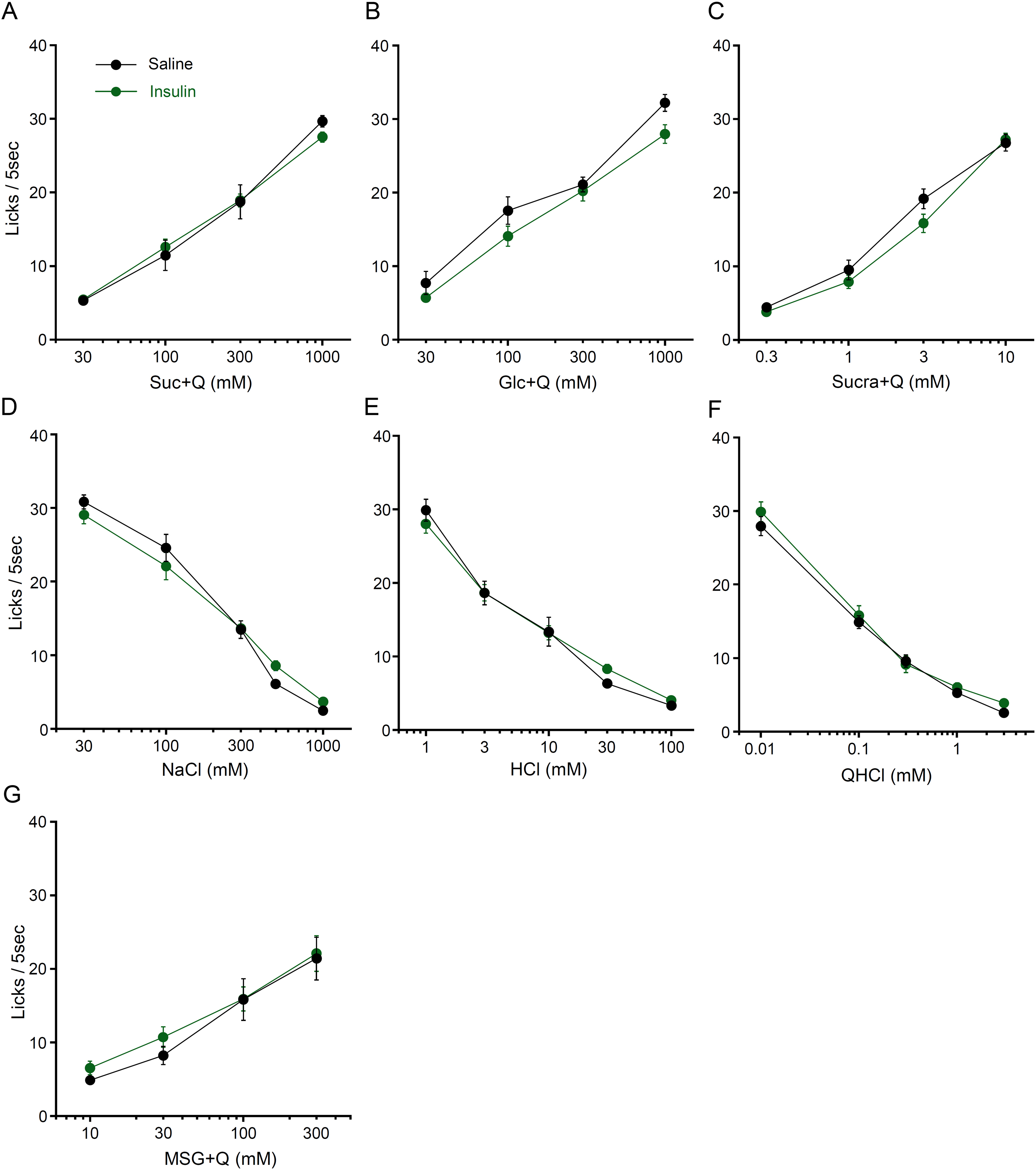

Supplement: Supplementary file 1 — Supplemental Figure 1. Licking responses to taste stimuli in short-term licking tests. Number of licking responses to 30–1000 mM Suc + Q (A), 30–1000 mM Glc + Q (B), and 0.3–10 mM Sucra + Q (C), 30–1000 mM NaCl (D), 1–100 mM HCl (E), 0.01–3 mM QHCl (F), and 10–300 mM MSG + Q (G). Green circles: Insulin group (0.1 mg/Kg body weight, n = 8), Black circles: Saline group (n = 7). Values are mean ± SEM [file mmc1.jpg]

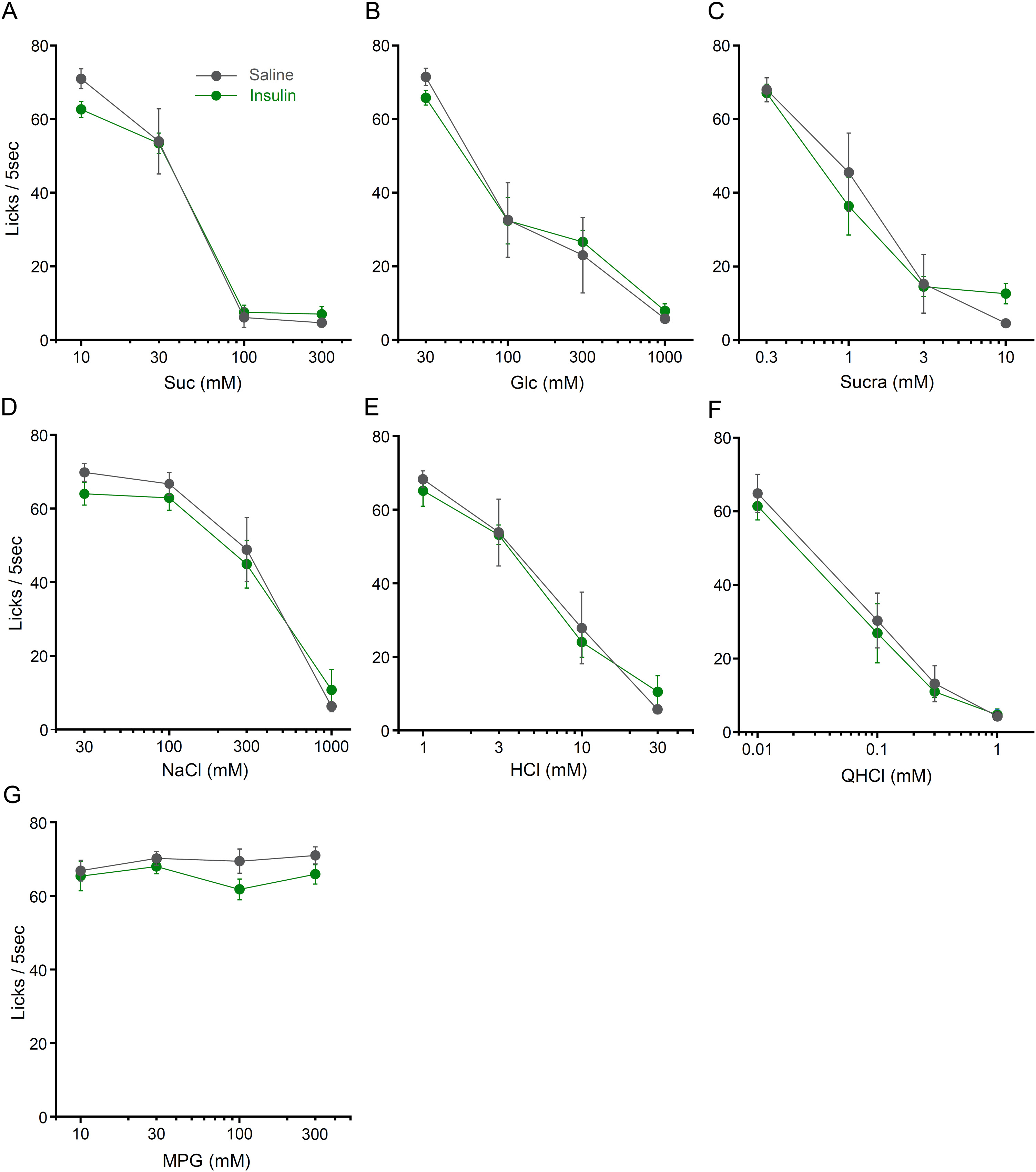

Supplement: Supplementary file 2 — Supplemental Figure 2. Licking responses to taste stimuli in CTA tests. Number of licking responses to 10–300 mM Suc (A), 30–1000 mM Glc (B), and 0.3–10 mM Sucra (C), 30–1000 mM NaCl (D), 1–30 mM HCl (E), 0.01–1 mM QHCl (F), and 10–300 mM MPG (G). Green circles: Insulin group (0.1 mg/Kg body weight, n = 8), Blue triangles: Black circles: Saline group (n = 7). Values represent mean ± SEM [file mmc2.jpg]
